# Recent advances in role of chromium and its antioxidant combinations in poultry nutrition: A review

**DOI:** 10.14202/vetworld.2016.1392-1399

**Published:** 2016-12-09

**Authors:** Z. Haq, R. K. Jain, N. Khan, M. Y. Dar, S. Ali, M. Gupta, T. K. Varun

**Affiliations:** 1 Division of Animal Nutrition, Faculty of Veterinary Sciences & Animal Husbandry, Sher-e-Kashmir University of Agricultural Sciences and Technology of Jammu, Jammu - 181 102, Jammu and Kashmir, India; 2 Department of Animal Nutrition, College of Veterinary Science & Animal Husbandry, Mhow - 453446, Madhya Pradesh, India; 3 Division of Instructional Livestock Farm Complex, Faculty of Veterinary Sciences & Animal Husbandry, Sher-e-Kashmir University of Agricultural Sciences and Technology of Jammu, Jammu - 181 102, Jammu and Kashmir, India; 4 Division of Veterinary Anatomy, Faculty of Veterinary Sciences & Animal Husbandry, Sher-e-Kashmir University of Agricultural Sciences and Technology of Jammu, Jammu - 181 102, Jammu and Kashmir, India; 5 Division of Animal Nutrition, National Dairy Research Institute, Karnal, Haryana, India

**Keywords:** antioxidants, chromium, poultry, stress

## Abstract

Poultry is reared in open side houses in most of the tropical countries, which results in huge temperature variation in shed causing stress resulting in increased demand of antioxidant supplementation. Since cooling of poultry houses or environment control is very expensive, thus methods focused on nutritional modifications appears to be the much logical approach. Stress increases mineral and vitamin mobilization from tissues and their excretion. Effect of some minerals and vitamin supplements such as chromium (Cr) and ascorbic acid to elevate the negative effects of environmental stress is well documented. Cr functions as an antioxidant and its deficiency are said to disrupt carbohydrate and protein metabolism. Cr has been utilized for weight gain, to improve feed conversion ratio, increase relative organ weight, muscle development, decrease cholesterol, increase high-density lipoprotein cholesterol, and improve nutrient digestion. Therefore, the present review discusses the beneficial aspects of Cr with its effect in different doses and antioxidant combinations to explore and promote its optimum utilization in poultry nutrition and production.

## Introduction

Metabolism of glucose in birds is considerably different from mammals as blood glucose concentration is much higher in birds and insulin levels are low [1]. Birds as compared to mammals are considered to be less sensitive to insulin [2] and effect of chromium (Cr) to enhance insulin sensitivity in mammals is well documented [3]. Heat or cold stress increases circulating concentrations of corticosterone in broilers and it is well documented that corticosterone reduces insulin sensitivity in broilers [4]. Poultry is reared in open side houses in most of the tropical countries like India, which results in huge temperature variation in the shed [5] causing stress which results in increased demand of antioxidant supplementation. Since cooling of poultry houses (environment control) is very expensive, thus methods are focused on nutritional modifications [6] like search of new feed additives along with their different combinations to increase the performance of birds naturally. Stress increases mineral and vitamin mobilization from tissues and their excretion [7], thus may exacerbate a marginal vitamin and mineral deficiency or an increased mineral and vitamin requirement. It has been reported that the negative effects of environmental stress could be prevented by the use of some minerals and vitamin supplements such as vitamin C and Cr [8,9]. Cr is essential for proper insulin action, is required for normal protein, fat, and carbohydrate metabolism, and is acknowledged as a dietary supplement in humans also [10]. Trivalent Cr is associated with the metabolism of carbohydrates, lipids, and proteins in animals termed as “glucose tolerance factor” since Cr regulates the metabolic action of insulin [11]. However, different Cr forms, i.e. organic and inorganic have diverse rates of absorption. Organic Cr (Cr-yeast) has greater biological availability than inorganic Cr. Among inorganic sources, the most common forms of Cr are the metallic form, Cr (0), trivalent Cr (III), and hexavalent Cr (VI). The hexavalent form is a known toxin, mutagen, and carcinogen [10,12]. The availability of inorganic Cr (e.g., CrCl_3_) is very low, in the range of 0.5-2%, whereas organic Cr (e.g., Cr Pic) is better, i.e., in the range of 10-25% [13]. Cr is said to improve weight gain, feed conversion ratio (FCR), increase relative organ weight, lean muscle development, reduce abdominal fat, decrease cholesterol, increase high-density lipoprotein (HDL) cholesterol, improve nutrient digestion, and elevate negative effects of environmental stress [14-21].

Although birds do not require any dietary vitamin source as it can synthesize vitamin C. Pardue and Thaxton [22] reported that particular environmental stressors could alter ascorbic acid utilization or synthesis in poultry. It is well documented that under stress conditions such as low or high environmental temperatures, humidity, and high productive rate ascorbic acid synthesis is inadequate [23]. Poultry cannot synthesize vitamin E; thus, vitamin E requirements must be met from dietary sources [24] in case of increased demand in stress. Vitamin E is a biological chain-breaking antioxidant that protects cells and tissue from lipoperoxidative damage induced by free radicals [23]. Sahin *et al*. [24] reported that broilers supplemented with dietary Cr and vitamin E significantly alleviated the heat stress related decrease in performance suggesting that additional supplementation into diets may be necessary under stress conditions in growing birds.

The present review discusses the beneficial aspects of Cr with its effect in different doses and antioxidant combinations to explore and promote its optimum utilization in poultry nutrition and production. So, it will be highly useful for scientists, researchers, veterinary professionals, poultry industry, pharmaceutical industry to enrich their knowledge in promoting Cr, and its antioxidant combination usage.

## Feed Intake and Feed Conversion

A plenty of literature suggests that supplementation of Cr at different levels and combinations in poultry improved feed intake and efficiency [16,25]. Kim *et al*. [26] revealed that 1600 or 3200 Cr Pic supplementation also improved feed efficiency without affecting feed consumption in broilers. Samanta *et al*. [27] reported 0.5 mg/kg of Cr to improve the feed intake and FCR. Lee *et al*. [28] conducted two trials to evaluate the effect of Cr on the performance of broilers and found Cr supplementation improved feed efficiency in broilers. Holoubek *et al*. [29] conducted an experiment in cockerels and pullets that received Cr in form of chromium picolinate (Cr Pic) at a dose 300 µg/kg. Results indicated that Cr-supplemented group of cockerels and pullets exhibited similar feed conversion, however, feed consumption per unit gain reduced in Cr-supplemented group. Debski *et al*. [30] observation revealed a slightly lower (statistically nonsignificant) feed consumption and feed conversion in the group receiving Cr-yeast. Kroliczewska *et al*. [31] evaluated the effect of Cr-enriched yeast supplementation on performance of broilers and concluded that supplementation of Cr either at 300 or 500 µg/kg of diet did not affect FCR up to 21 days but supplementation of 500 µg Cr/kg significantly improved FCR at 22-42 days of age. Furthermore, FCR of the entire trial (1-42 days of age) was reported higher in 500 µg Cr/kg diet supplemented group in comparison to 300 µg Cr/kg diet supplemented group and control diet.

Many studies support Cr supplementation in poultry diet to improve performance during heat or cold stress [8,24,32]. Supplementing Cr at dose of 1500 ppb to broilers in heat stress showed a positive effect on feed intake and feed efficiency [33]. Some studies have also found a negative effect of Cr on feed intake and FCR [34]. Amatya *et al*. [35] reported that supplementation of inorganic Cr (potassium chromate and Cr chloride) and organic Cr (Cr-yeast) at 0.2 mg Cr/kg feed significantly improved FCR of broiler birds in the 5^th^ week during summer in comparison to control.

There are studies which support synergistic action of Cr and other antioxidants during stress conditions, by sparing each other resulting in enhanced performance of birds [14]. Sahin and Sahin [8] evaluated the effect of ascorbic acid Cr Pic supplementation in laying hens reared under a low ambient temperature (6.2°C). It was concluded that a combination of 250 mg vitamin C and 400 µg Cr/kg of diet gave the best results in terms of feed consumption and FCR. Li *et al*. [36] conducted a study to evaluate the effect of Cr Pic acid and vitamin E alone and in combination on the performance of Cherry Valley ducks and found that Cr Pic acid and vitamin E supplementation lowered FCR in combination than Cr alone.

## Growth Performance

Cr supplementation increases the live weight gain and carcass yield in broilers [16]. According to other studies, Cr supplementation at different levels in nonruminants increases the carcass quality, weight gain, and organ weight with less carcass fat [16,37,38]. Kroliczewska *et al*. [31] conducted an experiment to evaluate the effect of Cr-yeast on performance of broilers. The experimental data revealed that supplementation of 500 µg/kg Cr either for 21 or 42 days significantly increased body weight and body weight gain as compared to the dose of 300 µg/kg Cr and control diet. Amatya *et al*. [35] conducted study on performance of broiler chickens exposed to natural heat stress during summer. They observed that during 5^th^ week only, the differences with respect to the live weight gain between control group birds and all Cr-supplemented experimental groups were found to be significant. Halder and Ghosh [20] observed that both final live weight and total live weight gain in 40 days improved in Cr-supplemented groups. Essa *et al*. [18] examined the effect of supplementing different levels of Cr, *viz*., 0.5, 1.0, 1.5, and 2.0 mg Cr-yeast/kg diet in broiler chickens. They inferred that live body weight and weight gain significantly increased in broiler supplemented with 0.5 and 1.0 mg Cr-yeast/kg feed at end of 3 weeks, whereas, at the end of 5^th^ week, high dosage of Cr-yeast supplementation (1.0, 1.5, and 2.0 mg Cr-yeast/kg feed) resulted in significantly increased live body weight and weight gain. However, there are studies which suggest that supplementation of Cr had no effect on body weight gain of broilers [15,30,39]. Luciana *et al*. [40] concluded that Cr tripicolinate had no effect on performance of broilers. Some studies on Cr at dose rate of 400 ppb Cr-yeast report a negative effect of Cr on body weight gain [34].

Many studies suggest that Cr performs better in terms of weight gain and carcass conformation in combination with antioxidants such as ascorbic acid or vitamin E [6,41,42], especially in case of stress conditions such as high or low temperature and humidity. Sahin *et al*. [17] conducted an experiment to study the effect of combination of Cr and vitamin E in cold stressed Japanese quails supplemented with either 400 µg of Cr/kg of diet (Cr group), 250 mg of α-tocopherol-acetate per kg of diet (vitamin E group), or 400 µg of Cr plus 250 mg of α-tocopherol-acetate per kg of diet (vitamin E + Cr group). Supplemental Cr and vitamin E significantly increased live weight gain, egg production, and improved feed efficiency in cold-stressed laying hens. However, a combination of vitamin E and Cr, rather than each separately, provided the greatest performance also increased serum vitamin C, vitamin E, and decreased malondialdehyde (MDA) concentrations. Mirfendereski and Jahanian [43] effects of dietary supplementation of Cr-methionine and vitamin C on performance of broilers and found that Cr-Met supplementation was more effective in improving egg production and FCR that unsupplemented diets. Ipek and Sahan [44] carried out a study to determine the effect of vitamin E and vitamin C alone, and in combination, on the performance of Japanese quails reared under heat stress and found that live weight was in chicks on a combination of 240 mg of vitamin E and 240 mg of vitamin C.

## Hematological Profile

### Glucose

Cr is biologically active as part of a biomolecule called chromodulin, which is part of an insulin signaling pathway thus affecting carbohydrate and lipid metabolism via the action of insulin [45]. Insulin has been shown to increase the glucose and amino acid uptake into muscle cells thus regulating energy production, muscle tissue deposition, and fat metabolism. In case glucose is not utilized by body cells due to a low insulin level, it gets converted to fat and stored in fat cells. Furthermore, if adequate amino acids cannot enter the cells, muscles cannot be built [46]. Cr is known to enhance insulin sensitivity in mammals and Cr supplementation has reduced plasma glucose concentrations in broilers [26,32,47].

Bakhiet and Elbadwi [48] on the basis of experimental findings reported that serum glucose values of broiler groups supplemented with inorganic Cr (Cr chloride) at the level of 0.2, 0.3, and 0.4 mg Cr/kg diet were significantly decreased as compared with control broiler group. Halder and Ghosh [20] observed on experimentation that measurement of serum glucose concentration significantly declined in the experimental broiler groups supplemented with 0.5 and 1 mg Cr/kg diet when compared with control group. Patil *et al*. [49] conducted research and observed that serum glucose concentrations were significantly and gradually reduced among broilers of treatment groups supplemented with organic Cr in the form of Cr Pic at level 200, 400, and 600 ppb/L through water than control. Luma *et al*. [19] observed that blood glucose of all chicken groups supplemented with 1 mg Cr/kg diet from inorganic (CrCl_3_) and organic (Cr-yeast and Cr-pic) were significantly decreased as compared with control group. Aslanian *et al*. [50] conducted a research to evaluate the effect of Cr methionine on performance and serum metabolite in growing-finishing male broiler and observed that starter and finisher period supplementation of Cr-Met decreased the blood concentrations of glucose significantly. However, there are some reports which show no effect of Cr on blood glucose [51].

### Serum triglycerides, cholesterol, HDL, and low-density lipoprotein (LDL)

In recent years, considerable research attention on the utilization of Cr in broiler diets has been given and several studies on the effects of Cr on cholesterol level and lipid profile have been published. In general, supplemental Cr has reduced the fat in serum and carcass as well [24,33]. Moeini *et al*. [32] concluded that Cr, especially in organic form reduces serum triglycerides and LDL cholesterol but elevates serum HDL cholesterol concentrations in heat-stressed broilers. Kim *et al*. [52] also reported increased HDL cholesterol, decreased LDL cholesterol, and higher ratios of HDL cholesterol in Cr Pic supplemented broilers. An increase of HDL cholesterol [53] and a decrease in total cholesterol and triglycerides [54] have been observed in humans after Cr supplementation. In previous studies, the beneficial effects of Cr on growth and blood parameters have been attributed to chromodulins ability to increase the sensitivity of tissue receptors to insulin [45]. Research studies suggest Cr involvement in carbohydrate metabolism including glucose uptake, glucose utilization for lipogenesis and glycogen formation [45,55].

Kroliczewska *et al*. [56] evaluated the effect of Cr from Cr-yeast on serum lipid profile of broiler chicken. They observed that serum total cholesterol and LDL significantly reduced, whereas serum HDL cholesterol significantly increased due to supplementation of 500 µg Cr/kg diet in broilers as compared to control. Bakhiet and Elbadwi [48] investigated the effect of dietary supplementation with increasing level at 0, 0.2, 0.3, and 0.4 mg Cr/kg and found that serum total cholesterol and LDL were significantly lowered in all Cr-supplemented group than control. However, serum HDL was significantly increased in all Cr-supplemented groups than control group of chicks of broilers. Haldar and Ghosh [20] observed that serum total cholesterol was significantly lowered in broiler group supplemented with 0.5 mg/Cr/kg diet from Cr tripicolinate when compared with control and 1 mg Cr/kg dietary supplemented broiler group. Patil *et al*. [49] conducted experiment in broilers and result of the experiment indicated that serum lipid profile in terms of total cholesterol and LDL cholesterol was significantly reduced in broiler of Cr-supplemented group, whereas, DHL cholesterol increased significantly. Zha *et al*. [57] conducted an experiment on broilers and reported that Cr-supplemented at 500 µg/kg diet from Cr Pic significantly decreased total cholesterol level in thigh muscle as against control. Dhia *et al*. [21] observed that blood total lipid was significantly lowered in dietary Cr-supplemented. The data on blood total cholesterol showed significant reduction in Cr (from Cr-yeast) and a trend of reducing serum triglycerides but statistically not important in supplemented group as compared to control. Luma *et al*. [19] observed that blood total lipid and LDL was reduced in groups which received dietary Cr. Navidshad *et al*. [58] conducted an experiment and results indicated that the 500 µg/kg level of organic Cr decreased the plasma cholesterol concentration in the finisher phase. The Cr concentration of 1000 and 500 µg/kg was more effective at grower and finisher phase. Aslanian *et al*. [50] observed that during starter and finisher phase of broilers, supplementation of Cr methionine decreased the blood concentrations of cholesterol and LDL, whereas HDL concentration was increased.

Several researches have reported that the combination of vitamin C and Cr caused more significant changes in lipid profile of broilers than either vitamin C or Cr alone and speculated about the synergistic action of vitamin C and Cr. It was seen that serum glucose and cholesterol concentrations decreased, whereas protein concentration increased when both dietary vitamin C also Cr were supplemented. Increasing concentrations of corticosterone were parallel to increases in serum glucose and cholesterol concentrations [17]. Through increasing the effectiveness of insulin, Cr also indirectly empowers the ascorbic acid transportation [59,60]. In addition to this, Cr is thought to be essential for activating certain enzymes and for stabilization of proteins and nucleic acids [61-63]. It has been recognized that insulin metabolism influences lipid peroxidation [64]. It is well known that hot climate increases MDA concentration as a lipid peroxidation indicator [65]. Tatli Seven *et al*. [66] found that plasma MDA level was significantly decreased in vitamin C group compared to the control group. Separately or in combination supplemental vitamin C and Cr resulted in a decrease in MDA concentration [67]. It has also been reported that serum glucose and cholesterol decreased, whereas total protein, albumin, globulin, calcium, and phosphorus concentrations increased when dietary Cr, Se, and vitamin C singly or in combination were supplemented. Similarly, Kutlu and Forbes [68] reported that vitamin C supplementation increased plasma protein, whereas blood glucose and cholesterol markedly decreased in heat-stressed broilers. A likely mechanism by which vitamin C causes a reduction in corticosterone concentration is through inhibitory effect of vitamin C on glucocorticoid synthesis, and it has been postulated that the improved performance of poultry results from a decrease in protein derived glucogenesis [23].

## Carcass Characteristics and Meat Lipid Profile

Cr is also a cofactor of insulin, promoting insulin activity [69], and enhancing amino acid uptake into muscular cells for protein synthesis [70]. Dietary Cr supplementation has been reported to have a positive effect on meat quality [35] and carcass traits of broiler chicks in natural [71] or heat stress condition [24]. Lambert and Jacquemin [71] reported insulin inhibits gluconeogenesis and depresses adipocyte lipolysis by reducing the activities of adenylate cyclase and hormone sensitive lipase. Amayta *et al*. [35] observed an increase of the protein level in muscles of broilers fed a diet supplemented with Cr in the form of Cr chloride or Cr-yeast. Samanta *et al*. [27] reported meat protein accretion improved in broiler fed organic Cr under heat stress condition. Furthermore, increasing in protein levels in the carcass and liver of broilers given Cr Pic were observed [26].

Kroliczewska *et al*. [31] conducted a study to evaluate the effect of Cr-yeast on carcass characteristics in broilers and found that it decreased cholesterol level in muscles. The largest differences were observed in breast muscles in the group fed 500 µg/kg Cr, where the content of cholesterol decreased by approximately 19%. Suksombat and Kanchanatwee [72] reported that supplementation of organic Cr reduced breast meat fat content and increased breast meat protein content. Total cholesterol and triglycerides were reduced by organic Cr supplementation. Supplementation with 200 and 400 ppb of both Cr-yeast showed the lowest total cholesterol. Anandhi *et al*. [15] reported that total cholesterol of breast and thigh muscle were significantly reduced in all treatment groups of broilers supplemented with organic Cr at level 250, 500, and 750 µg/kg diet as compared to control. Zha *et al*. [57] reported Cr Pic significantly decreased total cholesterol level in thigh muscle as against control. Lien *et al*. [47] reported that liver weight percentage relative to body weight was increased in Cr-supplemented broiler group at level 800 ppb Cr/kg diet. Tolmir *et al*. [73] conducted a research study which revealed that breast muscle yield was significantly increased in organic Cr-supplemented broiler group when compared with inorganic Cr-supplemented and control broiler group. Toghyani *et al*. [33] observed that dressing percentage significantly increased and abdominal fat significantly decreased in all Cr-supplemented broiler groups. Further, they found that liver and heart weight percentage relative to body weight was decreased in Cr-supplemented broiler groups, but there was nonsignificant difference when compared with control group. Tolmir *et al*. [73] reported that there was a significant increase in breast muscle yield and nonsignificant effect was observed in leg muscle yield of the organic Cr-supplemented group.

Since Cr, vitamin E, and vitamin C (postulated to be antioxidants) have a protective effect on pancreatic tissue against oxidative damage [23,74]; they may help pancreas to function properly including secretions of digestive enzymes, thus improving retention of nitrogen and fat metabolism. Thus, it can be suggested that these antioxidants might have a synergistic action to modify the carcass and lipid profile as well. Ali *et al*. [75] conducted a study on ascorbic acid and observed that dressing yield, breast meat, total meat, and wing meat were higher in supplemented broilers. Idown *et al*. [76] observed that ascorbic acid reduced up to 10% cholesterol of biceps femoris muscle and exerted a reduction of 12% cholesterol of serum on the supplemental dose of 100 ppb ascorbic acid. Chae *et al*. [77] conducted an experiment to study the effects of incremental levels of vitamin E on meat quality of broilers and concluded that vitamin E supplementation at higher levels was found beneficial for increased chicken meat quality. Sahin *et al*. [17] experiment conducted to evaluate the effects of Cr Pic and ascorbic acid supplementation on performance and carcass characteristics of broilers reported that supplementing a combination of vitamin C (250 mg/kg of diet) and Cr (400 mg Cr/kg of diet) may offer a potential protective management practice in preventing heat stress-related depression in performance and carcass characteristics of broiler chickens.

### Serum minerals

Sahin and Onderci [78] reported increased serum concentration of calcium (Ca), phosphorous (P), and potassium (K) and decreased the level of sodium (Na). Uyanik *et al*. [79] documented that feeding Cr did not affect serum Ca and P but increased magnesium (Mg) concentration at the level of 100 mg/kg of feed. Sahin and Sahin [24] noted that Cr supplementation in the form of Cr Pic (400 µg/kg of diet) improved the retention of minerals and decreased the excretion of Ca, P, Cr, nitrogen (N), zinc (Zn), and iron (Fe) in laying hens. Sahin *et al*. [41] observed that Cr supplementation reduced the excretion rate of Zn and Fe in Japanese quail. According to Ahmed *et al*. [37], the superior retention of Zn and copper (Cu) in the body may be due to the supplemental Cr which might have reduced urinary losses of these elements. Amatya *et al*. [35] found that Cu, Zn, Fe, and manganese (Mn) retention was better when Cr was supplemented in the feed of broilers in the form of Cr-yeast, whereas Uyanik *et al*. [41] reported that 20 ppm CrCl_3_ increased serum Ca and Mg in laying hens. Effects of different levels of organic and inorganic Cr (Cr chloride and Cr L-methionine) showed an increase in serum Cr and Zn concentrations but decrease in Cu contents [80]. Dietary Cr Pic and ascorbic acid supplementation showed increased concentrations of Fe, Zn, Mn, and Cr in laying hens at a low ambient temperature [24], while Cr Pic and vitamin C supplementation in heat-stressed laying hens increased Ca and P concentrations [80].

### Cr toxicity

The hexavalent form of Cr has toxic effects on birds as it promotes the early aging process, reduces hatching ability and effects liver also [81]. It also causes malformation or fetal death and leads to neural deformities. It has damaging effects on DNA and leads to mutation. It affects the function of gastrointestinal microflora on chronic exposure to high dosages [82]. It has the lethal effects on embryo and causes the defects in the development process leading to early chick mortality. It has the negative effect on chick growth and development [81]. Toxicity can be developed when there will be the excess amount of trace mineral added in the feed of the poultry that may lead to decrease the production parameters such as egg production and defective embryo development along with toxicities [83].

## Conclusion

Dietary Cr has useful effects on feed consumption, nutrient digestibility, growth, lipid profile, and carcass characteristics. It is an essential mineral element that plays an important role in livestock and poultry nutrition, and recently, it has gained public attention due to its well-established effect on reducing cholesterol level of meat. Cardiovascular diseases such as coronary heart disease and atherosclerosis in humans are the leading cause of death worldwide which are strongly related to dietary intake of cholesterol and saturated fat. Thus, decreasing intake of cholesterol and saturated fats will be a choice of our health conscious society that too without compromising a lot on eating habits of economically growing society. Thus lean meat production through chromium supplementation will be in vogue in future.. Abdominal fat in poultry is considered as waste, and unnecessary wastage of feed energy and Cr supplementation has very promising response according to various studies conducted. From the above literature, it can be clearly pointed out that Cr when combined with ascorbic acid has got synergetic action. While in combination with vitamin E such results were not seen. Thus, it can be inferred that Cr in combination with ascorbic acid offers best results.

## Authors’ Contributions

ZH and RK wrote the introduction, feed intake, feed conversion and Growth Performance. MK, MY and SA compiled Hematological Profile, Carcass Characteristics and Meat Lipid Profile. MG and TKV wrote Serum minerals, toxicity and conclusion. All authors read and approved the final manuscript.
